# Haemorrhagic Brain Metastasis From Malignant Pleural Mesothelioma: A Rare Case

**DOI:** 10.7759/cureus.30345

**Published:** 2022-10-16

**Authors:** Cleofina Furtado, Sachin Srivastava, Sanjeev Nayak, Changrez Jadun, Zafar Hashim

**Affiliations:** 1 Department of Diagnostic and Interventional Radiology, University Hospitals of North Midlands NHS Trust, Stoke On Trent, GBR

**Keywords:** computed tomography (ct), metastasis, cerebral, haemorrhagic, malignant pleural mesothelioma (mpm)

## Abstract

Malignant pleural mesothelioma (MPM) typically has a short median survival of only a few months from diagnosis, with death usually due to thoracic disease. This has led to the belief in the past that mesothelioma rarely has distant metastasis, with cerebral metastasis accounting for only 3%. The multiple cases of brain metastasis from MPM recorded so far were discovered after death at autopsy. This report describes a rare case of known malignant mesothelioma with distant haemorrhagic metastasis to the brain, reviews current literature about its metastatic potential to the brain and discusses prognosis and management. We also review the imaging evaluation in known MPM patients with suspected intracranial involvement and describe typical imaging findings of parenchymal brain metastasis on computed tomography (CT) and magnetic resonance imaging (MRI).

## Introduction

Malignant pleural mesothelioma (MPM) is a rare, highly invasive tumour that arises from the mesothelial cells of the pleura or peritoneum and is associated with a bad prognosis [1]. The liver, adrenal gland, kidney, and bone are the most common metastatic locations [2]. Although MPM is known to spread hematogenously, intracranial metastases are rare to the brain [3]. This is due to the short survival clinical course of MPM [4]. The multiple cases of brain metastasis from MPM recorded were discovered after death [5-7].

This is a rare case of known MPM with distant brain metastases, which encourages further radiological investigations in the presence of symptoms. The incidence of haemorrhagic brain metastasis from known MPM, histological features, treatment options, prognosis and imaging findings are also briefly discussed.

## Case presentation

A 67-year-old female patient presented to the emergency department with progressive bilateral lower limb weakness and unsteadiness for a few days, followed by a recent episode of loss of consciousness.

**Figure 1:**
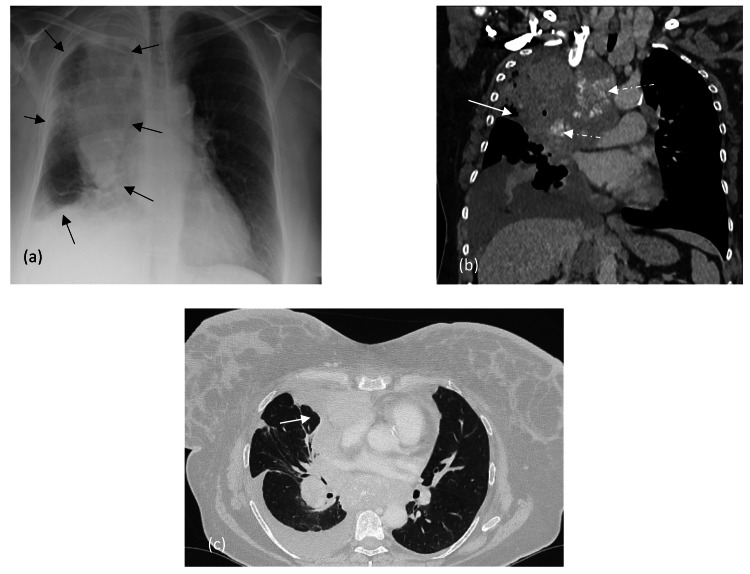
Chest radiographs (a): Circumferential pleural thickening of the right lung (within black arrows) with decreased lung volume. CT chest with contrast (b) and (c): nodular pleural thickening that encases the right lung with extension onto the mediastinal pleura (white solid arrows) and into the fissures. Large calcified pathologically enlarged mediastinal and hilar lymph nodes (white dashed arrows) are seen.

She was known to have malignant pleural mesothelioma (MPM) of the biphasic histologic subtype for which she underwent five cycles of chemotherapy (carboplatin and pemetrexed). It was discontinued due to toxicity and poor performance status, following which her disease progressed. The original pleural biopsy showed strong expression of Cam 5.2, MNF 116 and calretinin within epitheliod and spindle cells. CK5/6 was positive in the epitheloid tumour cell population, with WT-1 showing similar expression and additional focal expression in spindle cells. See Figure 1 for chest radiographs.

**Figure 2:**
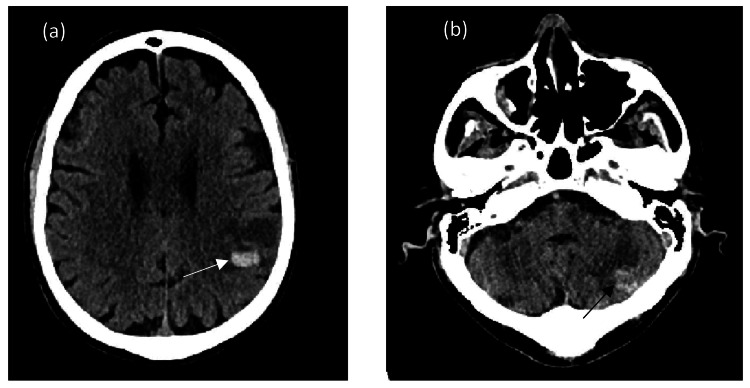
Plain axial CT demonstrating left posterior parietal (white solid arrow in (a)) and left cerebellar hemisphere (black arrow in (b)) with high attenuating haemorrhagic metastatic deposits and surrounding oedema

**Figure 3:**
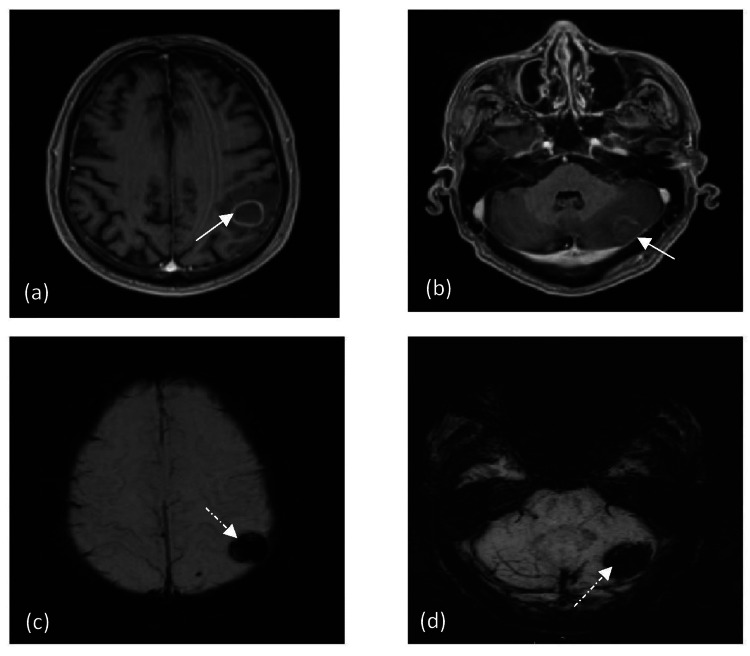
MRI brain T1WI axial contrast MRI (a and b) images depicting supra and infratentorial brain metastases with peripheral contrast enhancement and surrounding oedema (white solid arrows). Susceptibility weight imaging (SWI) (c and d) demonstrating corresponding blooming artefact (white dashed arrows) keeping with hemosiderin deposition.

Plain computed tomography (CT) head (Figure 2) performed for neurological impairment demonstrated two discrete supra and infratentorial hyperdense lesions with surrounding oedema. The subsequent magnetic resonance imaging (MRI) (Figure 3) demonstrated well-defined, intra-axial mass lesions in the left parietal and posterior fossa cerebellar hemisphere. It contained blood products with T1 hyperintensity on T1-weighted imaging (T1WI). The lesions depicted peripheral contrast enhancement with blooming artefact on gradient echo (GRE) sequence reflecting haemorrhagic nature.

Staging CT (chest, abdomen and pelvis) did not show any other synchronous primary tumour. Hence, a diagnosis of intracranial metastasis was made secondary to the known pleural mesothelioma. Given her primary malignancy progression, intolerance to chemotherapy and the multiplicity of brain metastasis, a discussion about prognosis and care was held with her and her family. The patient refused any aggressive treatment. Palliative radiation therapy and dexamethasone were administered in order to minimize oedema around the tumour and thereby relieve symptoms. The patient passed away eight months later.

## Discussion

Perineural spread, leptomeningeal carcinomatosis and, most commonly, haematogenous spread leading to parenchymal deposits are all possible mechanisms for central nervous system (CNS) metastases from MPM [8]. The clinical characteristics differ depending on the CNS area involved, with the cerebral cortex, cerebellum, intracranial meninges and spinal cord being the most commonly involved regions, and the midbrain, pons, or brainstem being less common [9].

In a retrospective analysis of 164 MPM patients, 67% were diagnosed with distant metastatic disease, with the highest frequency involving bone (19%), viscera (14%), contralateral lung (35%) and peritoneum (22%), and only (5%) with brain metastases [10]. A further case series from post-mortem examinations of 318 patients quantified brain metastases at 3% [11], whilst another systematic review reported a very low incidence of only 2.7% in autopsy patients [9].

The histologic appearance of MPM brain metastases may be comparable to glioblastoma multiforme [12]. There are three histological kinds of MPM: sarcomatous, epithelial and biphasic [2]. Patients with sarcomatous histology have the poorest prognosis, and conventional treatments, such as surgery or chemotherapy, are ineffective [13]. According to the existing research, in one review study, 11% of patients with cerebral metastases exhibited histological differences from the initial tumour, indicating histological differentiation into a more aggressive histological subtype [10]. 

CT is the first-line imaging modality in patients with new neurological impairments and known primary malignancy [14]. Acute haemorrhagic metastases to the brain appear hyperdense on non-contrast CT and are surrounded by varying vasogenic oedema [15]. Contrast-enhanced CT and MRI have higher sensitivity, demonstrating T1WI hyperintensity, depending on the age of haemorrhage associated with diffusion restriction and peripheral enhancement on contrast administration [15]. T2*-weighted GRE MRI is a sensitive tool for the early detection of metastases displaying haemorrhagic changes [16]. Our case demonstrates these typical features, indicating that brain metastases from MPM are typically haemorrhagic. Similar findings of brain metastasis with intratumoral haemorrhage secondary to MPM as a primary have been reported by Ishikawa et al. [3]. When haemorrhagic brain metastasis is the initial presentation, radiologists and clinicians should be aware of this pattern of brain metastasis and consider MPM as a differential when looking for the primary.

MPM with brain metastasis is considered a fatal disease [17], with most patients discharged without therapy or with only anti-oedema medication [18]. Other treatment options include resection, whole-brain radiation, stereotactic radiotherapy, systemic corticosteroids, intrathecal or systemic chemotherapy, and immunotherapy, but only a few instances have shown a therapeutic response [10]. Moreover, it is noted that brain metastasis can reappear within five to seven months following surgical or after regression in response to systemic treatment [3,19].

Local disease progression rather than distant metastasis is the most common cause of death [20]. The overall survival of patients with brain metastases is noted to be worse than that of patients without brain metastases with a mean survival of three months [3,18].

## Conclusions

MPM patients will continue to live longer as a consequence of newer, more advanced treatments, and hence more individuals will be diagnosed with metastatic disease. Therefore, it is critical to recognise mesothelioma's metastatic potential, particularly cerebral metastasis, which was previously only an autopsy finding. In patients presenting with neurological symptoms, haemorrhagic brain metastases should be considered as one of the differential diagnoses with known background history. At the same time, MPM should be included as a differential when brain metastasis with intratumoral haemorrhage is the initial presentation. The prognosis for malignant mesothelioma remains clinical and tumour histology-dependent.
